# Sea grapes extract improves blood glucose, total cholesterol, and PGC-1α in rats fed on cholesterol- and fat-enriched diet

**DOI:** 10.12688/f1000research.54952.1

**Published:** 2021-08-02

**Authors:** Mury Kuswari, Fahrul Nurkolis, Nelly Mayulu, Faisal Maulana Ibrahim, Nurpudji Astuti Taslim, Defny Silvia Wewengkang, Nindy Sabrina, Ghafur Rasyid Arifin, Keren Esther Kristina Mantik, Muhammad Rahimi Bahar, Najda Rifqiyati, Ronald Rompies, Piko Satria Augusta, Happy Kurnia Permatasari

**Affiliations:** 1 Nutrition, Universitas Esa Unggul, Jakarta, Jakarta, 11510, Indonesia; 2 Biological Sciences, State Islamic University of Sunan Kalijaga (UIN Sunan Kalijaga Yogyakarta), Yogyakarta, Yogyakarta, 55281, Indonesia; 3 Nutrition, Sam Ratulangi University, Manado, North Sulawesi, 95115, Indonesia; 4 Pharmaceutical Analysis and Medicinal Chemistry, Universitas Padjajaran, Sumedang, West Java, 45363, Indonesia; 5 Nutritional Sciences, Hasanuddin University, Makassar, South Sulawesi, 90245, Indonesia; 6 Pharmacy, Sam Ratulangi University, Manado, North Sulawesi, 95115, Indonesia; 7 Nutrition, Sahid University of Jakarta, South Jakarta, Jakarta, 12870, Indonesia; 8 Medicine, University of Indonesia, Depok, West Java, 16424, Indonesia; 9 Pediatrics, Sam Ratulangi University, Manado, North Sulawesi, 95115, Indonesia; 10 Medicine, Brawijaya University, Malang, East Java, 65145, Indonesia

**Keywords:** Caulerpa racemosa extract, blood glucose, total cholesterol, PGC-1α, functional food

## Abstract

**Background:** Sea grapes or  Caulerpa racemosa have a lot of phytochemical content, especially unsaturated fatty acids that are beneficial for health. This study aims to evaluate the effects of sea grapes extract on blood glucose levels, total cholesterol-, and Peroxisome proliferator-activated receptor-gamma coactivator (PGC)-1α in male Wistar rats, which were given per-oral (p.o.) cholesterol- and carbohydrates fat-enriched diets (CFED). **Methods:** Forty male Wistar albino rats weighing between 200 – 250 g were used for this study. Animals were randomly distributed into four groups of ten animals each. Group A served as control (received standard dry pellet diet). Rats in group B were fed on CFED for 4 weeks.  Groups C and D were fed on CFED and were administered 150 and 450 mg/kg of  sea grapes extract (p.o.), respectively. **Results:** Group C rats indicated a blood glucose reduction and an increase in PGC-1α serum, in comparison to group D (p<0.05). There were no significant differences between group C and D in blood cholesterol reduction (high dose of the extract did not have significant effects) (p=0.222), and both groups had the same effect in lowering total cholesterol in rats. **Conclusion:** Sea grapes extract is proven to improve blood glucose, total cholesterol, and PGC-1α levels in rats fed with CFED.

## Introduction

Reactive Oxygen Species (ROS) are the amounts of reactive molecules and free radicals derived from oxygen in a molecule ( *i.e.*, superoxide, peroxide, hydrogen peroxide, hydroxyl radical, *etc.*) ( Sies & Jones, 2020). Oxygen-based radicals are produced as a byproduct in the mitochondrial electron transport at the aerobic respiration performed by oxidoreductase enzymes and metal-catalyzed oxidation. A recent study has shown that ROS has a role in cell apoptosis that leads to organ dysfunction ( Pizzino et al., 2017).

Peroxisome proliferator-activated receptor-gamma coactivator (PGC)-1α is a transcription coactivator that regulates the genes involved in energy metabolism. It is the main regulator of mitochondrial biogenesis ( Liang &Ward, 2006). PGC-1α stimulates mitochondrial biogenesis and encourages the remodeling of muscle tissue to a fiber-type composition that is metabolically more oxidative and less glycolytic in nature, and it participates in the regulation of both carbohydrate and lipid metabolism ( Puigserver &Spiegelman, 2003; S. Yang et al., 2020).

The ability of cell defense against ROS has been associated with aging and contributes to the increased oxidative stress state. This condition can disturb the enzyme activity, especially through the reversible oxidative reaction at the thiol functional group at the side chain of the enzyme structures ( Birben et al., 2012). This can lead to the alteration of biomolecule structure and integrity, and enzyme dysfunction ( Freitas et al., 2016). As a result, insulin resistance and Type 2 Diabetes can development ( Facchini et al., 2001; Meigs et al., 2003). Additionally, the effect of aging on changes in liver mass can increase serum Low-Density Lipoprotein (LDL)-cholesterol level, due to the hepatocytes cell death caused by oxidative stress ( Anantharaju et al., 2002; Miller, 1984). Hence, effective control of ROS levels is essential. The aging population tends to have a higher prevalence of chronic disease, thus there is a demand for health-improving foods ( Park, 2013). The consumption and production of high-antioxidant as functional foods in recent years are popular due to their capability of reducing Reactive Oxygen Species (ROS), as well as having an impact on several aging and chronic related diseases ( Park, 2013; Park et al., 2004). However, there are some challenges that are associated with the utilization of functional food. For example, specific functional foods need to be consumed in high concentrations in order to be biologically effective, therefore, this would require the nutritional facts such as the daily dose of the bioactive compound in each serving size to be determined ( Kang et al., 2011). Preliminary studies are needed to determine which bioactive compound is the most beneficial, and what is the quantitative-activity relationship between the bioactive compounds contained in functional foods.

Sea grapes ( *Caulerpa racemosa*) or lawi-lawi (Indonesia-local terminology) is a species of editable green alga, seaweed in the *Caulerpaceae* family found in waters surrounding Sulawesi ( Pakki et al., 2020). Sea grapes are harvested intensively as they are an important source of macronutrients and micronutrients, especially in East and South-East Asia (grown commercially in ponds and consumed in the Philippines, Indonesia and Vietnam) as a major part of the traditional diet ( Chen et al., 2019). Some studies showed that sea grapes contains several bioactive components, such as protein, polysaccharides, polyphenol, flavonoids, and antioxidants (P. Yang et al., 2015; Yep et al., 2019; Taslim & Fahrul, 2021). Moreover, sea grapes have a high antioxidant level, and they have the potential to act as functional food or nutraceuticals ( Tanna et al., 2020; Yep et al., 2019; Nurkolis et al., 2021). The extract of sea grapes can reduce glucose level, aspartate aminotransferase (AST), alanine aminotransferase (ATL). Moreover, it appears to have a hepatoprotective activity in diabetic rats ( Qudus B Aroyehun et al., 2020). Therefore, this study aims to evaluate the effects of Sea grapes extract on blood glucose levels, total cholesterol, and PGC-1α in male Wistar rats on cholesterol- and fat-enriched diets (CFED).

## Methods

This *in vivo* study was conducted at the pharmacological laboratory, faculty of mathematics and natural sciences, Sam Ratulangi University.

## Collection and preparation of plant material

Fresh sea grapes ( *Caulerpa racemosa)* was collected from the shallow section (5-10 meters from the sea surface) of the Mantehage seawater, North Sulawesi, Indonesia. The botanical identification and authentication were confirmed at the department of pharmacology, faculty of mathematics and natural sciences, Sam Ratulangi University, Indonesia. The specimens were collected for feature references. The sea grapes were rinsed thoroughly with water, air-dried at room temperature and in an 40°C oven, then powdered by an electric mill.

## Preparation of sea grapes extracts

Crude powder (one kg) was macerated in 96% ethanol for 72 hours with each extraction performed in triplicates, which resulted in 34% yield. The crude extracts were filtered by Whatman 41 filter paper. The total filtrate was concentrated and evaporated at 40°C with a rotary evaporator RV 8 IKA under reduced pressure **(**100 millibar) for 90 minutes, and evaporated in an 40°C oven to produce a thick extract. The extract was stored in a refrigerator at 10°C until used in the study.

## Animal handling and ethical approval

All experimental rats were kept on standardized free access of feed and *ad libitum* of water. The study was conducted in the Laboratory of Pharmacology, Faculty of Mathematics and Natural Sciences, Sam Ratulangi University, Makassar, Indonesia. Forty male Wistar albino rats ( *Rattus norvegicus*) (4-5 weeks) weighing between 200 – 250 g were obtained from the Laboratory Animals Farming Makassar, Indonesia, and transported to the research site. The animals were grouped and housed in cages and maintained under standard laboratory conditions (temperature: 27 ± 2 ^o^C), with light and dark cycles (12/12 hours). The rats were acclimatized to laboratory conditions for 10 days before the commencement of the experiment. The research protocol (use of experimental animals) refers to the Declaration of Helsinki. The Council for International Organizations of Medical Sciences (CIOMS) has approved the application of ethical health research protocols online ( http://sim-epk.keppkn.kemkes.go.id) RSUP Prof. Dr. RD. Kandou, Manado with No. 086/EC/KEPK-KANDOU/VI/2021. Additionally, all experimental procedures were carried out according to the Institutional Animal Care and Usage Committee ( ARRIVE guidelines) (Nurkolis et al., 2021).

## *In vivo* studies of sea grapes extracts to evaluate blood glucose, total cholesterol, and PGC-1α levels

### CFED production

Carbohydrates fat-enriched diets (CFED) is a standard mouse food that comes with 1% colic acid, 2% pure cholesterol powder, 20% fat (animal source/pork oil), and 2% corn oil. Additional components are subtly added to the standard CFED and homogenized into a dough with the addition of 1000mL of aqua dest. Small pellets are cut and left to dry at room temperature in sterile conditions. CFED is prepared weekly and stored at 4°C until used to reduce oxidation. CFED consists of carbohydrate (43.57%), coarse protein (12.38%), coarse fiber (4.73%), coarse fat (3.17%), cholesterol (2%), colic acid (1%), animal fat (20%), corn oil (2%), total ash (4.3%), and moisture (6.85%). Compared to a normal diet that contains 58.1% carbohydrates, 16.51% coarse protein, and 0% animal fat, all the other components such as corn oil, cholesterol, and folic acid, were not significantly changed. CFED production guideline was carried out as previously described (Harb et al., 2018).

### Sea grapes extract administration scheme

Wistar albino rats were randomly distributed into four groups of ten animals each. Group A served as control (received standard dry pellet diet). Rats in group B were fed on CFED for 4 weeks. Rats in groups C and D were fed on CFED and were given 150 and 450 mg/kg Body Weight (BW) of sea grapes extract, respectively, for 4 weeks. CFED and extract of sea grapes were administered by oral gavage.

### Sample collection

Throughout the experiment, all the efforts were made to minimize the pain and distress of the experimental animals. For this purpose after four weeks of extract treatment, rats were kept fasted overnight and given euthanasia under ketamine anesthesia. 2.5 mL of blood samples were collected from the muscle tissue and kept in dry and clean tubes without addition of anticoagulants (Tiger-top tube), to allow clotting at room temperature. The samples were then centrifuged for 20 minutes at 3000 rpm. Finally, the sera were collected for the blood glucose, total cholesterol, and PGC-1α analysis.

### Biomedical analysis of blood sample

Blood glucose and cholesterol levels were assayed using COBAS Integra ^®^ 400 plus analyzer (Roche) ( See underlying data) ( Nurkolis, 2021). Samples were washed with Phosphate Buffered Saline (PBS, pH 7.4) 1% until the liquid runs clear. The samples were centrifuged at 3000 rpm for 20 minutes to obtain pellets and supernatant. The supernatant is taken for the PGC-1α examination ( See underlying data) ( Nurkolis, 2021). The concentration of PGC-1α was measured by using mouse PGC-1α ELISA Kit (Sunlong Biotech Co., Ltd, # MBS288117).

## Data management and analysis

The data were statistically analyzed with the use of the MANOVA/Multivariate ANOVA test. The Levene’s test was used to determine which posthoc tests should be conducted. In cases where the Levene’s test p-value was <0.05 Games-Howell test (equal variances not assumed), and for p-value >0.05 Bonferroni test (equal variances assumed) was used. Statistical analyses were performed by using SPPS 26.0 for the Windows version.

## Results

**Table 1.** Statistical interpretations based on homogeneity test.

		F	df1	df2	P-value
Glucose	Mean	10.495	3	36	.000 *
	Median	7.105	3	36	.001 *
	Median (adjusted df)	7.105	3	19.296	.002 *
	Trimmed mean	10.205	3	36	.000 *
Cholesterol	Mean	1.957	3	36	.138
	Median	1.741	3	36	.176
	Median (adjusted df)	1.741	3	23.800	.186
	Trimmed mean	1.853	3	36	.155
PGC-1α	Mean	9.042	3	36	.000 *
	Median	6.290	3	36	.002 *
	Median (adjusted df)	6.290	3	29.106	.002 *
	Based on trimmed mean	8.938	3	36	.000 *

F, Levene's Test; DF, Degrees of Freedom.

* Represents p-value <0.05, CI:95%.

The Levene's Homogeneity test shows that the p-value for glucose and PGC-1α are <0.05, therefore equal variance cannot be assumed, while equal variances can be assumed for cholesterol as the p-value is >0.05.

The results indicate that blood glucose significantly increased in group B, compared to group A (p < 0.05) ( Table 2). Blood glucose significantly decreased in both groups C and D (p < 0.05). The effect of sea grapes administration as much as 150 mg/kg BW is more effective than the sea grapes 450 mg/kg BW, in significantly decreasing blood glucose in rats (p < 0.05).

**Table 2.** The low dose of sea grapes is more effective in significantly reducing blood glucose.

	Diet	Mean	P-value
Group A	CFED	−15.3880	.000 *
	CFED + Sea grapes 150 mg/kgBW	5.9500	.000 *
	CFED + Sea grapes 450 mg/kgBW	2.8900	.001 *
Group B	Control	15.3880	.000 *
	CFED + Sea grapes 150 mg/kgBW	21.3380	.000 *
	CFED + Sea grapes 450 mg/kgBW	18.2780	.000 *
Group C	Control	−5.9500	.000 *
	CFED	−21.3380	.000 *
	CFED + Sea grapes 450 mg/kgBW	−3.0600	.003 *
Group D	Control	−2.8900	.001 *
	CFED	−18.2780	.000 *
	CFED + Sea grapes 150 mg/kgBW	3.0600	.003 *

* Represents p-value <0.05, CI: 95%.

As expected the rats in group B had significantly increased blood cholesterol levels compared to group A (p < 0.05). In both groups A and B (p < 0.05), blood cholesterol significantly decreased in rats given CFED + sea grapes extract 150 mg/kg BW, and CFED treatment + sea grapes extract 450 mg/kg BW. There was no significant difference between the CFED treatment group + 150 mg/kg BW sea grapes extract, and the CFED treatment group + 450 mg/kg BW sea grapes extract, in reducing blood cholesterol (high dose of the extract did not result in significant effects (p > 0.05)).

Group B had a significantly decreased PGC-1α serum concentration. PGC-1α serum concentrations significantly increased in group C, as well in group D, compared to groups A and B. The effect of sea grapes administration as much as 150 mg/kg BW is more effective than that of sea grapes 450 mg/kg BW, in the significant increase of PGC-1α serum in rats.

## Discussion

**Figure 1.**
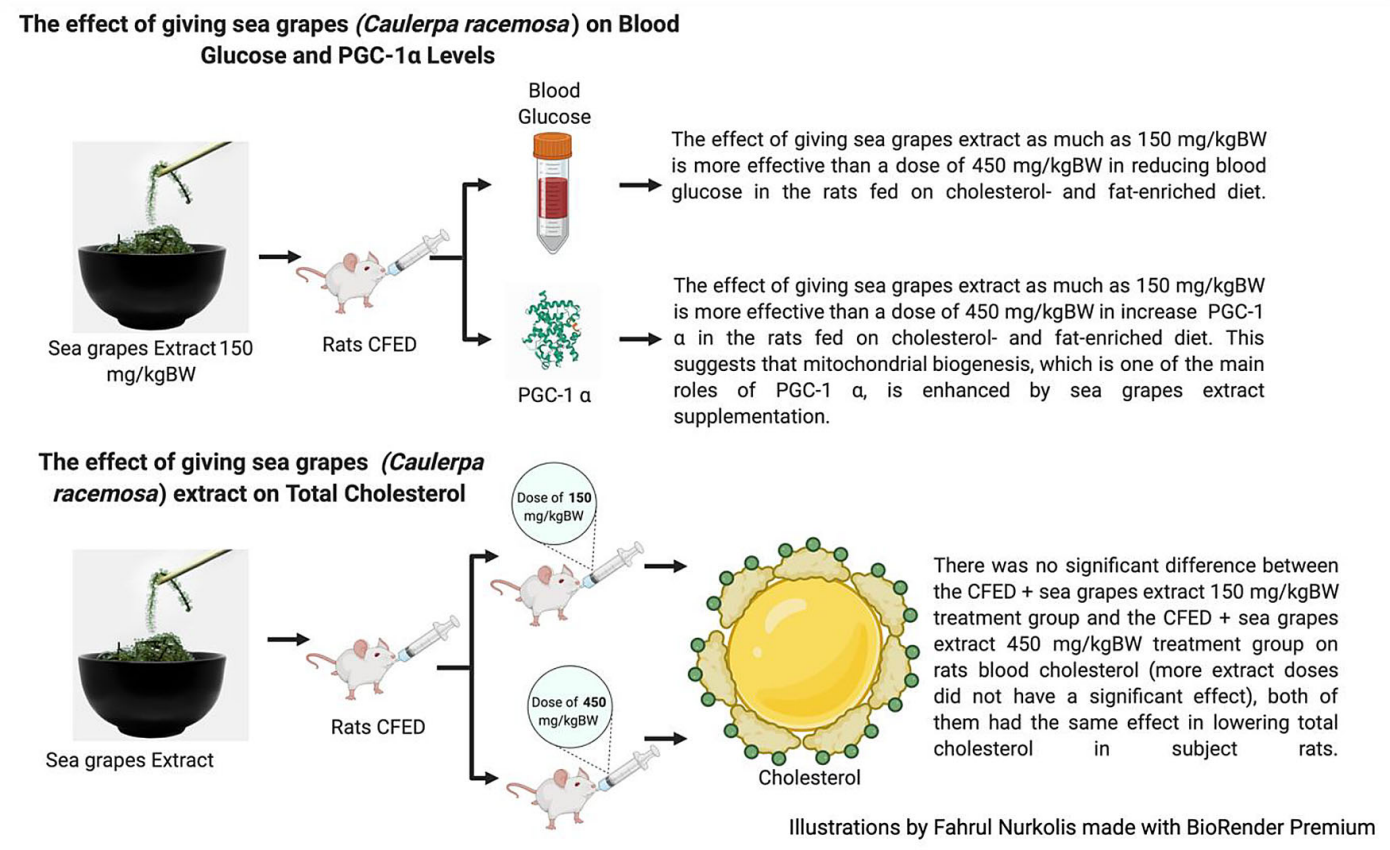
Effects of sea grapes extract on subject rats.

This study showed that the supplementation of sea grapes extract managed to lower blood glucose and serum cholesterol significantly in rats that were given cholesterol- and fat-enriched diets ( Figure 1). Although compared to the control group, rats that were given cholesterol- and fat-enriched diets with sea grapes extract had lower levels of blood cholesterol and blood glucose.

The Results of this study indicate that sea grapes have the capability of reducing blood glucose levels ( Table 2). Similarly, Aroyehun et al., have shown that sea grapes have antidiabetic activity. The plasma analysis in this study has also indicated that the sea grapes treated group had a significant decrease (p < 0.05) in their blood glucose levels compared to the untreated diabetic group ( Qudus B Aroyehun et al., 2020). Sea grapes extract-treated group demonstrated similar efficacy in lowering blood glucose as Metformin ( Qudus B Aroyehun et al., 2020), hence, sea grapes may have an hypoglycaemic effect. A hyperglycaemic state may induce oxidative stress that could be detrimental to insulin-sensitive tissues such as the liver, which may cause damage to the organ (Bugianesi 2005; Manna 2010; Palsamy 2010).

This study showed that sea grapes reduce hyperlipidemia in rats, however this is not in line with the findings by Aroyehun et al., ( Qudus B Aroyehun et al., 2020), which states that sea grapes extract has little to no effect on the cholesterol level of induced diabetic rats. In addition, the effect of lower doses of the extract (150 mg/kg BW) was better in lowering blood cholesterol than higher doses of sea grapes extract (450 mg/kg BW) ( Figure 1, Table 3). This can be due to the saturated fatty acids content, especially palmitic acid, which dominates the composition of fatty acids, comprising 80% of the total fat in sea grapes ( Qudus B Aroyehun et al., 2020). Studies have shown that palmitate acid may raise total cholesterol levels, specifically LDL-cholesterol levels ( Clandinin et al. 2000; Mensink, 2013).

**Table 3.** Both doses of sea grapes extract significantly reduce blood cholesterol.

	Diet	Mean	P-value
Group A	CFED	−22.1100	.000 *
	CFED + Sea grapes 150 mg/kgBW	12.0600	.000 *
	CFED + Sea grapes 450 mg/kgBW	8.0200	.001 *
Group B	Control	22.1100	.000 *
	CFED + Sea grapes 150 mg/kgBW	34.1700	.000 *
	CFED + Sea grapes 450 mg/kgBW	30.1300	.000 *
Group C	Control	−12.0600	.000 *
	CFED	−34.1700	.000 *
	CFED + Sea grapes 450 mg/kgBW	−4.0400	.222
Group D	Control	−8.0200	.001 *
	CFED	−30.1300	.000 *
	CFED + Sea grapes 150 mg/kgBW	3.0600	.003 *

* Represents p-value <0.05, CI: 95%.

Levels of PGC-1α in rats significantly decreased after being given a CFED diet compared to the control group ( Figure 1, Table 4). However, PGC-1α levels increased significantly in rats given sea grapes extract, even when compared to the control group. This suggests that PGC-1α, which is one of the major elements in mitochondrial biogenesis, is enhanced by the sea grapes extract. Perhaps the content of flavonoids as well as phenols in sea grapes extract can cause this effect. One study has shown that flavonoid supplementation increases the performance in endurance activities via an increase in expression of PGC-1α as the “master regulator” of biogenesis and skeletal muscle angiogenesis ( Khani et al. 2017). In addition, other studies have also shown that antioxidant compounds can upregulate PGC-1α target genes, which not only play a role in preventing oxidative damage, but also reduce mitochondrial ROS levels, ensure mitochondrial integrity during cell differentiation (Beldelli et al. 2014), as well as avoiding the cytotoxic effects of ROS accumulation ( St-Pierre et al. 2006).

**Table 4.** The low dose of sea grapes is more effective in significantly reducing PGC-1α.

	Diet	Mean	P-value
Group A	CFED	20.9200	.000 *
	CFED + Sea grapes 150 mg/kgBW	−19.3500	.000 *
	CFED + Sea grapes 450 mg/kgBW	−14.4200	.000 *
Group B	Control	−20.9200	.000 *
	CFED + Sea grapes 150 mg/kgBW	−40.2700	.000 *
	CFED + Sea grapes 450 mg/kgBW	−35.3400	.000 *
Group C	control	19.3500	.000 *
	CFED	40.2700	.000 *
	CFED + Sea grapes 450 mg/kgBW	4.9300	.000 *
Group D	control	14.4200	.000 *
	CFED	35.3400	.000 *
	CFED + Sea grapes 150 mg/kgBW	−4.9300	.000 *

* Represents p-value <0.05, CI: 95%.

## Conclusion

Sea grapes extract is proven to improve blood glucose levels, total cholesterol, and PGC-1α in rats fed with cholesterol- and fat-enriched diets. The results of this study can be used as a reference for clinical trials to further research the beneficial effects of sea grapes for human consumption. However, it is necessary to do the same research with parameters other than blood sugar, cholesterol and pgc-1a, to expand its metabolic scope.

## Data availability

### Underlying data

Harvard dataverse: Sea grapes extract effect on blood glucose level (BGL), total cholesterol (TC), and serum PGC-1α concentrations.

DOI: https://doi.org/10.7910/DVN/8IKREA ( Nurkolis, 2021).

The project contains the following underlying data:

• Raw data for the sea grapes extract effect on blood glucose level (BGL), total cholesterol (TC), and serum PGC-1α concentrations.

## Reporting guidelines

Harvard Dataverse: Arrive checklist for Sea grapes extract with blood glucose, total cholesterol, and PGC-1α in rats fed on cholesterol- and fat-enriched diet.

https://doi.org/10.7910/DVN/NXF0IW ( Nurkolis et al., 2021).

Data are available under the terms of the Creative Commons Zero “No rights reserved” data waiver (CC0 1.0 Public domain dedication).

## Author contributions

M.K and F.N. collated study ideas, designed and experiment, analyzed data, and compiled manuscripts. N.A.T, N. R, N. S, H.K.P, D.S.W and N. M analyzed and interpreted the data and critically revised the manuscript. F. N and F.M.I conducted experiments, analyzed biochemistry, and critically revised the manuscript. N. M, M.R.B, R. R, P.S.A and K.E.K.M, implemented experimental protocols, assisted in statistical analysis, interpreted data, and critically revised manuscripts. All writers read and approve the final manuscript.
