# Supplementary material for: Sea grapes extract improves blood glucose, total cholesterol, and PGC-1α in rats fed on cholesterol- and fat-enriched diet
Source: F1000Res. 2021 Sep 13;10:718. Originally published 2021 Aug 2. [Version 2] doi: 10.12688/f1000research.54952.2 (PMC8804902; doi:10.12688/f1000research.54952.2)
Supplement: Peer review history

# Peer review: Reviewer response for version 2

**DOI:** 10.5256/f1000research.77279.r110064

**Published:** 2022-01-31

**Authors:** Achuthan Raghavamenon

**Affiliations:** 1 Department of Biochemistry, Amala Cancer Research Centre, Thrissur, Kerala, India

In this study, the authors aim to see the effect of sea grape extracts as a function food on H fat cholesterol fed rats and observed that it improves blood sugar, cholesterol, and PGC-1α. The experimental plan is good and the results collected and analysed look fine. The conclusion derived seems overwhelming, as they collected only very few information. The following demerits have to be addressed:

1. The introduction describes much about oxidative stress, antioxidants, and flavonoid contents of Sea grape, however, no correlating information has been collected in the study.
2. The parameters studied in this manuscript are blood cholesterol, glucose and PGC1-alpha. As per the manuscript title, this article describes the improvement in the above mentioned parameters in rats fed a high cholesterol diet and then treated with sea grape extract. Why did the authors study only these three parameters? How are glucose level, cholesterol and PGC-1 alpha inter-connected? The introduction should discuss all this, so as to reflect a clear logic of the study.
3. Authors have given references for the anti-diabetic and cholesterol lowering effect of Sea grapes. Authors have not attempted to collect any information on triglycerides, HDL cholesterol, LDL cholesterol, etc. Also, what additional advantage blood PGC-1α provides is not clear.
4. The basis of the doses used in this study has not been adequately explained. Has any toxicity study been conducted before to arrive to these doses?
5. Authors have not done any histology of the liver to make sure that there is lower lipid deposition. There is the chance that lowering of blood lipids may be due to the accumulation of lipids in the liver tissue.
6. Tables 2, 3, and 4 have ABC & D groupings, and in each group, there is Control, CFED,CFED +150 mg extract AND cfed+ 450 extract. This is confusing. I suggest to make these tables direct values.
7. Spelling and grammar errors have to be addressed, for example, the use of present tense in the results section at some places, and use of past tenses at some other places. Such minor corrections can be checked and corrected by the authors while revising the manuscript.

Is the work clearly and accurately presented and does it cite the current literature?

Yes

If applicable, is the statistical analysis and its interpretation appropriate?

Yes

Are all the source data underlying the results available to ensure full reproducibility?

Partly

Is the study design appropriate and is the work technically sound?

Partly

Are the conclusions drawn adequately supported by the results?

Partly

Are sufficient details of methods and analysis provided to allow replication by others?

Yes

Reviewer Expertise:

Biochemistry , oxidized lipids and degenerative diseases

I confirm that I have read this submission and believe that I have an appropriate level of expertise to confirm that it is of an acceptable scientific standard, however I have significant reservations, as outlined above.

# Peer review: Reviewer response for version 2

**DOI:** 10.5256/f1000research.77279.r94240

**Published:** 2021-11-08

**Authors:** Chinedum Ogbonnaya Eleazu

**Affiliations:** 1 Department of Chemistry, Biochemistry and Molecular Biology, Alex Ekwueme Federal University, Ndufu-Alike, Ikwo, Nigeria

The article by Kuswari *et al.* has some interesting points. However, the manuscript will require revision by the authors for it to be worthy of indexing.

 General comments:

- The authors did not satisfactorily address the comments of the reviewers on their choice of dose of Sea grape extract they used for this study. Was an acute toxicity done to show the safety of the doses of this plant as used in this study? Again, there are several errors in the usage of English Language in this manuscript.

Specific comments:

 Abstract:

- L14, what do the authors mean by PGC-1α serum? Or do they mean in the serum? which is a better expression.

Introduction:

- Paragraph 3, suggest to correct 'Type 2 Diabetes development' to 'type 2 diabetes can develop or occur'. The continued sentence, 'additionally, the effect of ageing changes in the liver mass can increase serum low density lipoproteins........' is useless to the manuscript as ageing population was not a factor of interest in the manuscript.
- Paragraph 4, L8 of the introduction, if Sea grapes have been scientifically proven to reduce glucose level, why investigate it again in this study?
- This introduction needs to be re-written with the focus on what the authors determined, and their rationale for determining them. 

Collection and identification of plant material:

- The identification number of the plant is missing and should be reported.

Animal handling and ethical approval:

- The statement, 'the study was conducted in the laboratory........' is a repetition. 
- The carrier for the administration of the Sea grape extract should also be indicated. 
- Why did the authors use up to 10 animals per group? Did they subject their sample size to power testing before the study? The study appears overpowered.

Results:

- Table 1 is not cited in the text and the data on it needs to be explained. The title for table 2 should be changed to blood glucose concentrations of rats. Then, the effect of sea grape extract on blood glucose concentrations of the rats can be written as part of the results. 
- The unit of glucose is missing in the data presented in Table 2. Same is applicable for the units of cholesterol and PGC-1α in Tables 3 and 4. Why are some results in negative values? Authors need to revisit these data.
- Finally, in their blood sampling, authors indicated they worked with the sera of the animals, yet in different parts of the manuscript and in the results section, they reported their results on glucose and cholesterol as blood glucose and blood cholesterol. This should be revisited as well.

Is the work clearly and accurately presented and does it cite the current literature?

Partly

If applicable, is the statistical analysis and its interpretation appropriate?

Partly

Are all the source data underlying the results available to ensure full reproducibility?

Yes

Is the study design appropriate and is the work technically sound?

Partly

Are the conclusions drawn adequately supported by the results?

Partly

Are sufficient details of methods and analysis provided to allow replication by others?

Yes

Reviewer Expertise:

Diabetes, Antioxidants, Nutraceuticals, Metabolic syndrome

I confirm that I have read this submission and believe that I have an appropriate level of expertise to confirm that it is of an acceptable scientific standard, however I have significant reservations, as outlined above.

# Peer review: Reviewer response for version 2

**DOI:** 10.5256/f1000research.77279.r94138

**Published:** 2021-09-30

**Authors:** Carla F. Kairupan

**Affiliations:** 1 Bagian Patologi Anatomi Fakultas Kedokteran, Universitas Sam Ratulangi, Manado, Indonesia

The authors have addressed the comments that I made. I therefore think the revisions are appropriate.

Is the work clearly and accurately presented and does it cite the current literature?

Yes

If applicable, is the statistical analysis and its interpretation appropriate?

Yes

Are all the source data underlying the results available to ensure full reproducibility?

Yes

Is the study design appropriate and is the work technically sound?

Yes

Are the conclusions drawn adequately supported by the results?

Yes

Are sufficient details of methods and analysis provided to allow replication by others?

Yes

Reviewer Expertise:

Pathology, Molecular Genetics, Herbal Medicine

I confirm that I have read this submission and believe that I have an appropriate level of expertise to confirm that it is of an acceptable scientific standard.

# Peer review: Reviewer response for version 1

**DOI:** 10.5256/f1000research.58483.r90945

**Published:** 2021-08-20

**Authors:** Carla F. Kairupan

**Affiliations:** 1 Bagian Patologi Anatomi Fakultas Kedokteran, Universitas Sam Ratulangi, Manado, Indonesia

This is an interesting research, in which the treatment with Sea grapes extract clearly improved blood glucose levels, total cholesterol, and PGC-1α in rats. However, it is not the first study that is done on the beneficial effects of Sea grapes extract *.*

 The paper is well written and the experimental design is set up well, however, the statistical analysis should be better described.

 The following points should also be addressed appropriately in the revision.

1. The Laboratory of Pharmacology, Faculty of Mathematics and Natural Sciences, Sam Ratulangi University where the study was conducted is not in Makassar.
2. The terminology and composition of diets are inconsistent and unclear.
3. The area where the blood sample was collected is inappropriate.
4. The doses of Sea grapes extract were 150 mg/kg BW and 450 mg/kg BW. How did you decide on these doses? Are there any safety issues?

The article may also benefit from an English language edit.

Is the work clearly and accurately presented and does it cite the current literature?

Yes

If applicable, is the statistical analysis and its interpretation appropriate?

Yes

Are all the source data underlying the results available to ensure full reproducibility?

Yes

Is the study design appropriate and is the work technically sound?

Yes

Are the conclusions drawn adequately supported by the results?

Yes

Are sufficient details of methods and analysis provided to allow replication by others?

Yes

Reviewer Expertise:

Pathology, Moleculer Genetics, Herbal Medicine

I confirm that I have read this submission and believe that I have an appropriate level of expertise to confirm that it is of an acceptable scientific standard, however I have significant reservations, as outlined above.

# Response

**Published:** 2021-08-31

**Authors:** Fahrul Nurkolis

**Affiliations:** The State Islamic University of Sunan Kalijaga (UIN Sunan Kalijaga Yogyakarta), Indonesia

Dear Dr. Carla F Kairupan, PhD.,

 Thank you for the appreciation and constructive review comments to improve this paper.

 We have made minor improvements as you mentioned in the review, as follows:

 1. The Laboratory of Pharmacology, Faculty of Mathematics and Natural Sciences, Sam Ratulangi University where the study was conducted is not in Makassar, **it should be a typo and we have changed it to Manado.**

 2. The terminology and composition of diets are inconsistent and unclear. **We've made it clear.**

 3. The area where the blood sample was collected is inappropriate.

**We've made that clear, in the tail of the rats**.

  4. The doses of Sea grapes extract were 150 mg/kg BW and 450 mg/kg BW. How did you decide on these doses? Are there any safety issues? **In existing studies, most use 100 mg and 200 mg, therefore the choice of this dose is higher and different from previous studies and 450 is the maximum capacity for a safe rats stomach.**

 Thank you,

 Greetings, hope you are well!
